# Novel and Re-emerging Respiratory Viral Diseases: Novartis Foundation Symposium 290

**DOI:** 10.3201/eid1506.090293

**Published:** 2009-06

**Authors:** David M. Morens

**Affiliations:** National Institutes of Health, Bethesda, Maryland, USA

**Keywords:** Respiratory diseases, viruses, influenza, book review

This slim booklet, the product of a Novartis Foundation symposium held April 23–27, 2007, at Singapore’s Institute of Molecular and Cell Biology, primarily highlights scientific issues concerning influenza and severe acute respiratory syndrome (SARS). The booklet comprises a mere 12 chapters of about 12 pages each of reports presented and discussed by only 29 participants. This may be its strength—the book’s usefulness derives from its focus on only 2 diseases covered by multidisciplinary participants in a cross-cutting fashion and relative depth.

The book contains little new material. Rather, the chapters are brief but state-of-the-art reviews that, perhaps surprisingly, fit together well. For example, Gabriele Neumann and Yoshi Kawaoka discuss broad aspects of pandemic influenza; other scientists discuss the transmission and pathogenicity of influenza viruses A (H5N1), their genetic and antigenic characteristics, general antigenic associations between human and swine viruses, and the molecular aspects of viral membrane fusion. Reading for less than half an hour yields an awareness of many key issues surrounding influenza emergence, well reviewed by international experts. The chapters about SARS are equally strong, with presentations by teams from both Taiwan and Singapore highlighting their real-world experiences in an epidemic crisis. More general presentations by Larry Anderson and Suxiang Tong (characterization of novel viruses) and by Malik Peiris and Yi Guan (the animal–human interface) provide excellent background and balance. A particularly interesting and strong chapter by Eddie Holmes reviews viral evolution and emergence, analyzing viral host-switching and the theoretical frameworks used to study it.

The chapters are all well written, well edited, succinct, and readable, apparently aimed at scientists—readers familiar with both research and public health aspects of emerging viral diseases. A bonus is inclusion of discussions by the participants at the end of each chapter. Although the value of these varies, in many cases the discussion provides additional perspectives that otherwise would be missed.

The book is a pleasant surprise: modest, succinct, authoritative, readable, and enjoyable. It is particularly valuable for scientists and advanced students who either work with the 2 diseases in question or who work with issues of viral disease emergence.

